# Evidence of association between the use of drugs and community-dwelling older people frailty: a cross-sectional study

**DOI:** 10.1590/1516-3180.2020.0205.R1.06082020

**Published:** 2020-10-09

**Authors:** Marcos Kaic Lopes Alves, Nayara Gomes Nunes Oliveira, Maycon Sousa Pegorari, Darlene Mara dos Santos Tavares, Maria Cristina Soares Rodrigues, Alisson Fernandes Bolina

**Affiliations:** I BSc. Pharmacist, Universidade de Brasília – Campus Darcy Ribeiro (UnB), Brasília (DF) Brazil.; II PhD. Nurse, Universidade Federal do Triângulo Mineiro (UFTM), Uberaba (MG), Brazil.; III PhD. Physiotherapist and Adjunct Professor, Physiotherapy Course, Universidade Federal do Amapá (UNIFAP), Macapá (AP), Brazil.; IV PhD. Nurse and Associate Professor, Department of Nursing Education and Community Health Nursing Undergraduate Program, Universidade Federal do Triângulo Mineiro (UFTM), Uberaba (MG), Brazil.; V PhD. Nurse and Pharmacist, Associate Professor, Universidade de Brasília – Campus Darcy Ribeiro (UnB). Brasília (DF), Brazil.; VI PhD. Nurse and Adjunct Professor, Universidade de Brasília – Campus Darcy Ribeiro (UnB), Brasília (DF), Brazil.

**Keywords:** Polypharmacy, Drug interactions, Potentially inappropriate medication list, Older people, Frail older people, Polypharmacy among older people

## Abstract

**BACKGROUND:**

The scientific literature has shown that an association between polypharmacy and frailty exists. However, few studies have also considered drug interactions and the use of potentially inappropriate medications.

**OBJECTIVE:**

To evaluate the association between the use of drugs and frailty among community-dwelling older people.

**DESIGN AND SETTING:**

Cross-sectional study carried out among 580 older people in Uberaba (MG).

**METHODS:**

Data were collected at these older people's homes using instruments validated in Brazil. Descriptive, bivariate and binary logistic regression analyses were performed (P < 0.05).

**RESULTS:**

Most of these individuals were classified as pre-frail (55.7%), while 13.1% were frail. It was found that 31.7% of them presented polypharmacy, 41.7% had drug interactions and 43.8% were using potentially inappropriate medications. In the initial model, polypharmacy (odds ratio, OR = 1.91; confidence interval, CI = 1.27-2.86) and use of potentially inappropriate medications (OR = 2.45; CI = 1.68-3.57) increased the chance that these older people would be pre-frail or frail. In the final adjusted model, use of potentially inappropriate drugs remained associated with the outcome (OR = 2.26; CI = 1.43-3.57).

**CONCLUSION:**

Use of potentially inappropriate medications was the independent variable that explained the occurrence of frailty in a representative sample of community-dwelling older adults.

## INTRODUCTION

Frailty syndrome among older people is related to changes that occur through the human aging process, such as sarcopenia, neuroendocrine dysregulation and immune system dysfunction.^1^ Frail individuals are at increased risk of adverse events and injuries due to falls, which, together with various comorbidities, can cause higher rates of institutional care, hospitalizations and mortality.^1^^,^^2^

The aging process can promote physiological changes that cause older people to exhibit distinctive pharmacokinetics, such that they may become more sensitive both to the therapeutic effects and to the toxic effects of drug therapy.^3^ Furthermore, multimorbid conditions require the use of multiple drugs, which is characterized as polypharmacy. This, together with the physiological changes of aging can increase the chances of adverse events among older people.^3^^–^^6^ These include the increased levels of pathogenesis within frailty syndrome, as highlighted in the International Frailty Consensus.^7^

There are several concepts of polypharmacy, although most of them consider it to be the concomitant use of five or more drugs.^8^ This was the concept used in the present investigation. It is important to note that polypharmacy increases the risk of drug interactions (DI), as well as the use of potentially inappropriate medications (PIMs) among older people.^3^ Polypharmacy, therefore, cannot be considered to be the only marker for assessing the quality of drug prescriptions,^9^ which requires consideration of DIs and use of PIMs for clinical care among older people.

DIs consist of clinically significant changes to the effect of a given drug caused by administration of another drug. Such changes may lead to modification of the absorption capacity to bind to proteins, or of the metabolic or excretion rate of one or even two of the medications involved in the interaction concerned.^10^^,^^11^ Faced with considerable increases in the proportion of drug prescriptions issued to older people and the consequent increased risk of adverse events among these individuals, there is concern regarding identification and prevention of undesirable combinations and use of PIMs.

It is known that PIMs increase the chances of adverse outcomes among older adults and that these are exacerbated when frailty syndrome is present.^9^^,^^13^^,^^14^ Nevertheless, studies in the scientific literature on this topic have focused on demonstrating the association between polypharmacy and frailty,^15^^,^^16^ but without including evaluations of DIs and PIMs. It is also worth mentioning that older people, including frail individuals, experience reduced efficacy of medications, in addition to higher risk of adverse effects.^17^ The possible explanations for this phenomenon include impaired physiological systems that combat frailty, drug interactions, drug-disease interactions and reduced adherence to medication. Additionally, adverse reactions to medications go unnoticed and can lead to other prescriptions.^17^

The existence of this gap in knowledge emphasizes the need for clarifications regarding the relationship of these variables with frailty syndrome among community-dwelling older people. Better knowledge of the implications arising from variables relating to use of drugs can improve preventive clinical approaches towards the embrittlement process among older people. This could lead to significant differences in quality of life during the aging process.

## OBJECTIVE

The objective of this study was to evaluate the association between the use of drugs and frailty among community-dwelling older people.

## METHODS

### Design

This cross-sectional study consisted of a household survey conducted among older people living in the urban area of the city of Uberaba, Minas Gerais, in the southeastern region of Brazil. This study followed the guidelines of the Checklist for Reporting Results of Internet E-Surveys and the guidelines for Strengthening the Reporting of Observational Studies in Epidemiology (STROBE).

### Sample

The sample size calculation considered a prevalence of frailty of 12.8%,^6^ accuracy of 2.7% and a 95% confidence interval for a finite population of 36,703 older people. From this, the sample size was determined as 579 subjects. However, allowance was made for a sampling loss of 20% and therefore it was calculated that the maximum number of individuals to be approached would be 724 elderly people. To define the study population, a multistage cluster sampling process was used, considering census tracts, with information on neighborhoods and streets provided by the Brazilian Institute for Geography and Statistics. Tracts were drawn in order to subsequently select older people living in these tracts.

Older adults aged 60 or older, who were living in the urban area of the municipality and who were able to walk, were included in the study. It needs to be highlighted that, in Brazil, people aged 60 years or over are considered to be older adults, according to the current legislation.^18^

Subjects were excluded from this study in the following situations: presentation of cognitive decline, as assessed using the Mini Mental State Examination (MMSE);^19^ failure to locate the individual after three visits; hospitalization and/or institutionalization; and inability to undergo the assessment of frailty. This assessment because impossible if the subject presented inability to walk, severe sequelae from stroke, localized loss of strength and aphasia, or a severe or unstable stage of Parkinson's disease and associated severe impairment of motility, speech or cognition.

In the end, a total of 768 older people were approached, taking into account both the inclusion criteria and the losses, which comprised 154 due to cognitive decline and 34 due to incomplete tests for frailty evaluation. Hence, 580 patients were assessed in the present study.

### Data collection

The interviews took place in the older people's homes, in the period from March to June 2016. They were conducted by trained interviewers with previous experience in collecting data. Five supervisors, who had previously been selected, checked the interviews to verify the filling out and consistency of the items, in order to ensure quality control.

### Explanatory and adjustment variables

The explanatory and adjustment variables were collected using a structured questionnaire that sought the following information: (1) socioeconomic: age (numerical variable) and/or age group in years (60 to 69, 70 to 79 and 80 or older); gender (male or female); marital status (with or without a partner); schooling, in years (no education, 1 to 4 years and 5 years or more); individual monthly income, in minimum wages (no income, ≤ 1 minimum wage and > 1 minimum wage); and (2) number of self-reported morbidities (0, 1 to 4 and 5 or more), as described in a previous study.^20^

### Frailty syndrome (dependent variable)

Presence of frailty syndrome, which was taken to be the dependent variable, was identified through the five items that were proposed as components of the frailty phenotype by Fried et al.:^1^

1. Unintentional weight loss: assessed through the question: “In the last year, did you lose more than 4.5 kg without intention (that is, without dieting or exercise)?”.
2. Self-report of exhaustion and/or fatigue: assessed through two questions from the Brazilian version of the Center for Epidemiological Studies (CES-D) depression scale, i.e. item 7 (“Did you feel you had to make an effort to cope with your usual tasks?”) and item 20 (“Were you unable to carry on with your things?”). The elderly people with a score of 2 or 3 in either of these questions met the frailty criterion for this item.^21^
3. Decreased muscle strength, as assessed from handgrip strength using a manual hydraulic dynamometer (Model SH5001, SAEHAN, São Paulo, Brazil) and adopting the cutoff points proposed by Fried et al.^1^
4. Slow gait speed, obtained from the gait time (in seconds) that was needed to cover a distance of 4.6 meters, using the cutoff points proposed by Fried et al.^1^
5. Low level of physical activity, as ascertained from the long version of the International Physical Activity Questionnaire (IPAQ), adapted for older people.^22^ The classification used for this component considered older people to be inactive if they had 0-149 minutes of physical activity per week.^23^

The older people who were positive for three or more of these items were classified as frail and those who were positive for one or two items were classified as pre-frail. Those who were negative in all the tests were considered to be robust or non-frail.^1^

### Drug use (independent variables)

To assess the variables relating to drug use, the older subjects were first asked: “Could you show me the medications you are currently using?” Thus, they were asked about their medical prescriptions and the packaging of the drugs that were being used at the time of data collection. The following were recorded: the pharmaceutical form of the medicinal products, the amounts consumed and the number of applications per day. Based on these data, situations of polypharmacy, DIs and PIMs were evaluated, as described below.

Polypharmacy was checked by counting the number of medications used by each older individual. When these older people reported using five or more medications, they were deemed to present polypharmacy.^8^

Occurrences of DIs were also assessed through the Micromedex Drug Reax System (Greenwood Village, Colorado, USA), using its online access platform,^24^ which contains evidence-based information on drugs and diseases. This tool allowed identification of the DIs that occurred (drug-drug) and ranked them according to severity (severe, moderate or mild). It is worth noting that this tool is widely recognized worldwide for use by healthcare professionals, including pharmacists, to obtain unbiased data. The value of this tool has been sustained through systematic reviews on the subject.^24^

Use of PIMs was classified in accordance with the criteria established in the Brazilian Consensus on Potentially Inappropriate Drugs for Older People.^12^ To analyze this variable, the subjects were divided between: “Using PIMs”, when it was found that they were using at least one drug classified as inappropriate; and “Not using PIMs” when they did not use any of these drugs.

### Data analysis

The data were entered into an electronic spreadsheet in the Excel software, in duplicate, in order to identify any possible inconsistencies from data entry. Subsequently, the data were imported into the Statistical Package for the Social Sciences (SPSS) software, version 22.0 (New Orchard Road, Armonk, New York, USA), to carry out the analyses.

A descriptive statistical analysis was conducted by distributing absolute and percentage frequencies. The bivariate analysis on the socioeconomic characteristics and variables relating to use of drugs according to frailty condition was done using the chi-square test. To assess associations among use of polypharmacy, PIMs and DIs in relation to the frailty syndrome, the logistic regression model was adopted. In this model, the outcome variable was recategorized so as to become dichotomous (frail/pre-frail versus non-frail). In the final adjusted model, the independent variables were included (polypharmacy, use of PIMs and DIs), along with other potential confounding variables such as gender, age, education and number of self-reported morbidities. For all analyses, the tests were considered significant when P < 0.05.

### Ethical considerations

This study was approved by the human-research ethics committee of the Federal University of Triângulo Mineiro (Universidade Federal do Triângulo Mineiro, UFTM), under protocol no. 493,211, dated December 13, 2013.

## RESULTS

Out of the total number of participants (n = 768), 154 older people were excluded because they presented cognitive decline and 34 because of inability to perform the comprehensive evaluation of the components of the frailty phenotype. Thus, the final sample consisted of 580 older adults.

In comparing the older people who were excluded with those who participated in the study, it was found that for both groups, the majority were female (70.7% versus 68.1%; P = 0.418); were living without a partner (71.3% versus 52.4%; P = 0.353); had had one to four years of schooling (56.4 versus 52.6%; P = 0.352); had a monthly income of less than or equal to two minimum salaries (88.2% versus 81.6%; P = 0.979); and had five or more self-reported morbidities (64.7% versus 62.4%; P = 0.493). Regarding the age groups, older people aged 70 to 79 years (30.9%) predominated among the excluded individuals; while older adults aged 60 to 69 years (44.1%) predominated among those who participated in the study. However, there was no significant difference regarding age groups (P = 0.645).

Based on the final sample (n = 580), the frailty status among the subjects was as follows: 13.1% (n = 76) were frail; 55.7% (n = 323) were pre-frail; and 31.2% (n = 181) were non-frail.

It was found that most of the participants were female (68.1%); were between 60 and 69 years old (44.1%); were living without a partner (52.4%); had had one to four years of schooling (52.6%); and had a monthly income of two minimum wages (46.0%), followed by ≤ 1 minimum wage (44.7%). In analyzing the sociodemographic variables according to the frailty classification, a higher percentage of older people aged 70 to 79 years (P < 0.001) and with no education (P = 0.008) was observed in the frail and pre-frail groups, compared with the non-frail group. It was also observed A higher proportion of older people with five or more frail and pre-frail morbidities was also observed, in relation to the non-frail ones (P = 0.013) (Table 1).

**Table 1:** Absolute and percentage frequency distributions of the sociodemographic and health variables of the elderly subjects, according to their frailty phenotype classification; Brazil, 2016

Variables		Frailty phenotype			Total % (n)	P*
		Non-frail % (n)	Pre-frail % (n)	Frail % (n)		
**Gender**	Male	30.4 (55)	34.4 (111)	25.0 (19)	31.9 (185)	0.252
	Female	69.6 (126)	65.6 (212)	75.0 (57)	68.1 (395)	
**Age group (in years)**	60 to 69	59.1 (107)	39.3 (127)	28.9 (22)	44.1 (256)	< 0.001
	70 to 79	35.4 (64)	42.4 (137)	38.2 (29)	39.7 (230)	
	80 or older	5.5 (10)	18.3 (59)	32.9 (25)	16.2 (94)	
**Marital status**	Companion	48.1 (87)	52.6 (170)	47 (61.8)	304 (52.4)	0.130
	No companion	51.9 (94)	47.4 (153)	29 (38.2)	276 (47.6)	
**Education (years of schooling)**	No education	11.6 (21)	16.1 (52)	23.7 (18)	15.7 (91)	0.008
	1 to 4	47.5 (86)	55.7 (180)	51.3 (39)	52.6 (305)	
	5 or more	40.9 (74)	28.2 (91)	25.0 (19)	31.7 (184)	
**Monthly income**	No income	11.6 (21)	9.0 (29)	5.3 (4)	9.3 (54)	0.132
	≤ 1 minimum wage	39.8 (72)	44.6 (144)	56.6 (43)	44.7 (259)	
	> 2 minimum wages	48.6 (88)	46.4 (150)	38.2 (29)	46.0 (267)	
**Number of morbidities**	0	4.4 (8)	1.3 (4)	0 (0)	2.1 (12)	0.013
	1 to 4	37.6 (68)	36.8 (119)	25.0 (19)	35.5 (206)	
	5 or more	58.0 (105)	61.9 (200)	75.0 (57)	62.4 (362)	

* Chi-square test.

**Figure 1:**
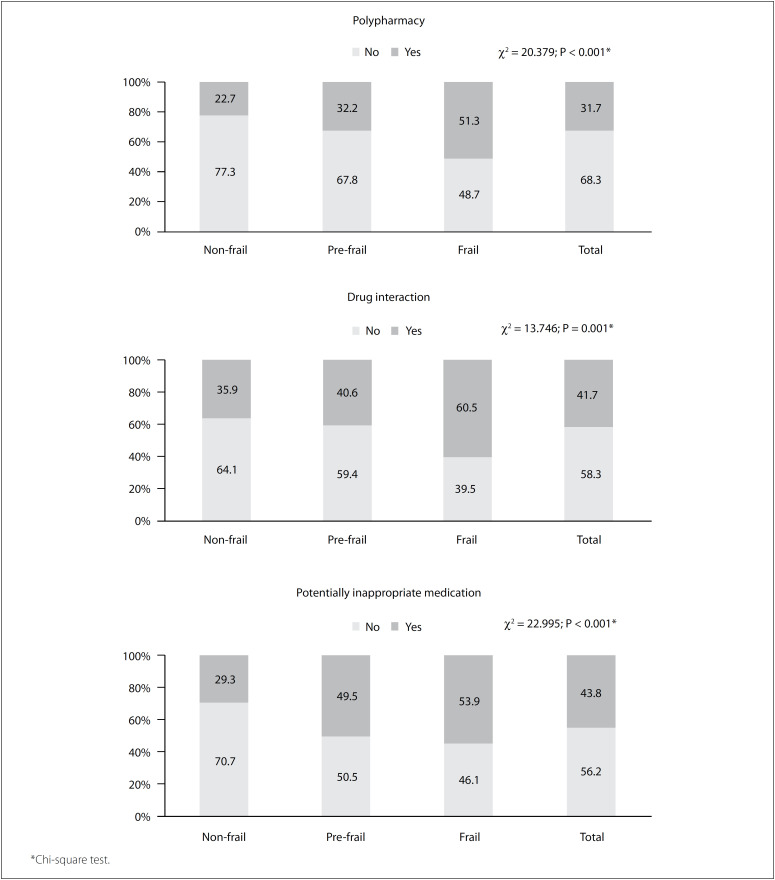
Occurrences (%) of polypharmacy, drug interaction and use of potentially inappropriate medication for elderly people, according to the frailty phenotype classification; Brazil, 2016.

Presence of polypharmacy was found in 31.7% (n = 184) of the older people. It was found that 41.7% (n = 242) had at least one DI and 43.8% (n = 254) were using PIMs. Occurrence of these events was more common among the frail older people, among whom 51.3% (n = 39) presented polypharmacy (P < 0.001), 60.5% (n = 46) had DIs (P = 0.001) and 53.9% (n = 41) had PIM use (P < 0.001), in comparison with the other groups (Figure 1).

In logistic regression analysis, it could be seen in the initial model that presence of polypharmacy (odds ratio, OR = 1.91; confidence interval, CI = 1.27-2.86) and use of PIMs (OR = 2.45; CI = 1.68-3.57) increased the odds of occurrence of frailty/pre-frailty among these community-dwelling older people. Evaluation of the final adjusted model showed that use of PIMs remained associated with increased chances of occurrence of frailty/pre-frailty (OR = 2.33, CI = 1.47-3.70), regardless of gender, age group, number of self-reported morbidities, education or other variables relating to use of medications (polypharmacy and DIs). It is noteworthy that age was also an explanatory variable for occurrences of frailty (Table 2).

**Table 2:** Logistic regression models to verify associations among polypharmacy, drug interaction and use of potentially inappropriate medications in relation to the elderly frailty phenotype, Brazil, 2016

		Pre-frail/frail		Pre-frail/frail	
		Initial model		Adjusted final model	
		OR	CI	OR	CI
**Polypharmacy**	No	1	1	1	1
	Yes	**1.91** ‡	**1.27-2.86** ‡	1.23	0.69-2.19
**DI**	No	1	1	1	1
	Yes	1.42	0.99-2,04	0.77	0.46-1.28
**PIM**	No	1	1	1	1
	Yes	**2.45** ‡	**1.68-3.57** ‡	**2.33** ‡	**1.47-3.70** ‡
**Gender**	Female	−	−	1	1
	Male	−	−	0.79	0.52-1.21
**Age**	−	−	−	**1.08** ‡	**1.05-1.11** ‡
**Education**	No education				
	1 to 4 years			0.79	0.58-1.04
**Number of diseases**	−	−	−	1.04	0.98-1.11

Reference category = non-frail;

‡ P < 0.001; 1 = reference category.

OR = odds ratio; CI = confidence interval; DI = drug interactions.

## DISCUSSION

The data from this study highlight that frailty among older people is a serious public health problem, given that significant prevalence (13.1%) of this event among elderly individuals living in their own homes was demonstrated. This finding was similar to what has been found in other studies conducted in Brazil and worldwide that also used Fried's phenotype: 12.8%,^6^ 14.8%,^25^ 10%^26^ and 14%.^27^ However, it differed from others that have identified higher prevalences (47%^28^ and 65.25%^29^) through using the Tilburg Frailty Indicator (TFI) concept and instrument.

This divergence of results was expected, given that the prevalence of frailty may vary according to the diagnostic instrument, methodological standardization, plurality of existing concepts and variability of sample composition.^30^ A systematic review by Collard et al. showed that there was marked variation in the prevalence of frailty among community-dwelling older people, from 4.0% to 59.1%.^31^ These data emphasize the need for these differences to be considered not only by healthcare professionals in evaluating older people within clinical practice but also by managers in formulating public health policies.^31^

Since drug prescription is a participant in the frailty process, its quality requires special attention from healthcare professionals. The aging process makes older people more susceptible to developing chronic conditions, which eventually leads to use of several medications concomitantly.^9^ This, together with the pharmacokinetic and pharmacodynamic changes that occur with advancing age, results in exacerbated adverse effects, especially when the frailty syndrome is present.^9^^,^^32^

These results converge with findings that were highlighted by other researchers, through demonstration of the association between polypharmacy and frailty in the initial logistic regression model.^15^^,^^16^^,^^33^ According to the International Frailty Consensus, polypharmacy is a possible cause of increased pathogenesis of frailty. Hence, reduction of the use of drugs for older people is recommended, among other clinical guidelines.^7^ A longitudinal study on Japanese older people found that those who used six or more drugs were at higher risk of developing frailty, in relation to the others, over a six-year period.^34^ It is worth considering, however, that polypharmacy did not remain associated with an increased chance of frailty in the adjusted model of the present study, and this was also seen in other studies.^29^^,^^35^ These data highlight the importance of including other variables associated with evaluation of the quality of drug use among older people within clinical practice.

The relationship between DI and frailty was also analyzed in the present study but no significant association was found, either in the initial logistic regression model or in the adjusted model. Pagno et al. found that 52.2% of the older people were exposed to DIs, which was a result similar to that of the present study. They also found that most older people with DIs were classified as frail (68.2%) and demonstrated that exposure of older people to DIs increased the chance of this outcome. However, they did not carry out multivariate analysis with adjustment for other variables.^33^ It is important to note that most of the researchers who have evaluated DIs among older people did not consider frailty to be a factor associated with this event, as seen in an integrative review of the literature conducted by Rodrigues and Oliveira.^3^ Hence, there is a need for further clarification of this relationship through additional studies.

In the current study, use of PIMs was the independent variable that explained the increased chances of occurrence of frailty, thus confirming other findings that have been described in the literature.^33^^,^^36^^–^^39^ The hypotheses that might contribute towards understanding this association include the following:

- Use of PIMs can worsen older people's clinical state, thereby interfering with their quality of life and increasing the magnitude of adverse health outcomes;^12^^,^^33^ and these occurrences are exacerbated when frailty syndrome is present.^9^^,^^13^^,^^14^
- Among the adverse outcomes relating to use of PIMs, a strong association with functional decline has been shown;^36^ this is significantly correlated with frailty syndrome, as shown by Fried et al.^1^
- PIMs can affect the components that are measured in the frailty phenotype, such as weakness, low gait speed or low levels of physical activity.^1^^,^^36^

The findings of the current study demonstrated that use of PIMs was highly prevalent among these community-dwelling older people and that its presence was associated with occurrences of frailty. These findings are concordant with the clinical guidance for management of frailty, in which reduction or deprescription of potentially inappropriate medication for older adults is strongly recommended.^40^ Curtin et al. used the STOPPFrail criteria and demonstrated that this is a tool that removes an important barrier against deprescription of medications through explicitly highlighting the circumstances in which commonly used medications can be safely deprescribed among older people with advanced frailty.^41^

Professionals working within primary healthcare are in closer contact with community-dwelling older people and, therefore, should incorporate evaluation of use of PIMs in their overall routine for geriatric assessment. In this regard, the International Frailty Consensus recommends use of the Beers and STOPPFrail criteria within clinical practice. When use of PIMs is identified, the older individuals presenting this usage should be referred for medical evaluation, in order to optimize their medication treatment and, therefore, prevent frailty syndrome. Lavan et al. found that almost 65% of their patients awaiting long-term care were eligible for application of the STOPPFrail criteria, such that over 90% of these had been prescribed at least one PIM. They concluded that the transition to nursing-home care represented an opportunity to review the therapeutic appropriateness and goals of the medications that had been prescribed for these individuals.^42^

Although use of PIMs was the only explanatory independent variable for frailty syndrome in the present study, the importance of evaluating polypharmacy and DIs cannot be overlooked. It is known that both the presence of PIM and the presence of polypharmacy tend to make frail older people more prone to negative events, such as increased risk of adverse effects, mostly coming from DIs. These relationships can be explained in terms of the changes and features present in frail older people that make them more vulnerable to manifestations of DIs and health problems arising from them.^33^^,^^43^^,^^44^

In addition, several studies have shown that use of multiple medications is associated with use of PIMs.^38^^,^^45^^–^^51^ Other authors have shown, however, that the risk of using PIMs is greater among individuals with higher numbers of morbidities and who, thus, have to use more drugs.^48^^,^^52^^,^^53^ Pagno et al. also identified that the prevalence of frailty was higher in the presence of PIMs that were involved in DIs.^33^ Moreover, Lorenzo-López et al. confirmed the dynamics of frailty and the bidirectional nature of frailty transitions, thus indicating the need for prevention and treatment of these conditions in later life, in order to minimize the burden of frailty.^54^

The findings from the present study need to be considered cautiously due to its cross-sectional nature, which did not allow cause-and-effect relationships to be established among the variables. Moreover, it needs to be borne in mind that a self-report questionnaire was used to investigate morbidities, which meant that some of the information found may have been underestimated or overestimated. Therefore, use of cohort studies among community-dwelling older people is suggested, in order to assess the effect of interactions among the variables of DI, PIM and polypharmacy, regarding occurrences of frailty syndrome.

## CONCLUSION

It was found that use of inappropriate medications was the independent variable that explained the occurrences of frailty in this representative sample of community-dwelling older people in a Brazilian municipality. However, this study showed that there is a need for research with a longitudinal design, in order to assess the causality of these conditions in relation to frailty.

Nevertheless, the data obtained in this study constitute an advance in this field of knowledge, since they indicate the need for advanced practices, with application of explicit methods for evaluation of drug use within primary healthcare, with a view to improving the quality of life of older people living in their own homes. Thus, in clinical practice, accurate analysis with the use of validated tools and technologies for monitoring and recognition of polypharmacy, potential drug interactions and inappropriate use of drugs can optimize the adequacy of prescription and hence minimize problems relating to these medications, thereby diminishing the onset of frailty.
